# The Effect of Feed Supplementation with EM Bokashi® Multimicrobial Probiotic Preparation on Selected Parameters of Sow Colostrum and Milk as Indicators of the Specific and Nonspecific Immune Response

**DOI:** 10.1007/s12602-021-09850-z

**Published:** 2021-10-01

**Authors:** Łukasz Jarosz, Artur Ciszewski, Agnieszka Marek, Zbigniew Grądzki, Beata Kaczmarek, Anna Rysiak

**Affiliations:** 1 Department of Epizootiology and Clinic of Infectious Diseases, Faculty of Veterinary Medicine, University of Life Sciences in Lublin, Głęboka 30, 20-612 Lublin, Poland; 2 Sub-Department of Preventive Veterinary and Avian Diseases, Faculty of Veterinary Medicine, Institute of Biological Bases of Animal Diseases, University of Life Sciences in Lublin, 20-950 Lublin, Poland; 3 Department and Clinic of Animal Internal Diseases, Sub-Department of Internal Diseases of Farm Animals and Horses, Faculty of Veterinary Medicine, University of Life Sciences in Lublin, Głęboka 30, 20-612 Lublin, Poland; 4 Department of Botany, Mycology, and Ecology, Maria Curie-Skłodowska University, Akademicka 19, 20-033 Lublin, Poland

**Keywords:** Sow, Colostrum, Milk, Flow cytometry, Acute phase proteins, Lysozyme

## Abstract

The aim of the study was to determine the effect of EM Bokashi® on selected parameters of the specific and nonspecific immune response of sows by in colostrum and milk samples. The percentage of cells with expression of CD19^+^, CD5^+^CD19^+^, CD21^+^, SWC3a (macrophage/monocyte), and CD11b^+^ molecules on the monocytes and granulocytes as well as the concentrations of lysozyme and acute phase proteins — serum amyloid-A (SAA) and haptoglobin (Hp) were evaluated. The study was carried out on a commercial pig farm, including 150 sows (Polish Large White × Polish Landrace) at the age of 2–4 years. Sixty female sows were divided into two groups: I — control and II — experimental. For the experimental group, a probiotic in the form of the preparation EM Bokashi® in the amount of 10 kg/tonne of feed was added to the basal feed from mating to weaning. The material for the study consisted of colostrum and milk. The samples were collected from all sows at 0, 24, 48, 72, 96, 120, 144, and 168 h after parturition. The study showed that exposure of the pregnant sow to the probiotic microbes contained in EM Bokashi® significantly affects the immunological quality of the colostrum and milk and caused an increase in the percentage of the subpopulations of B cells with CD19^+^, CD21^+^, and CD5^+^CD19^+^ expression in the colostrum and milk, which demonstrates an increase in the protective potential of colostrum and indicates stimulation of humoral immune mechanisms that protect the sow and the piglets against infections.

## Introduction

Intensive pig farming is based mainly on the effort to increase the productivity of sows [1]. Breeding work and achievements in genetics exploited in pig breeding currently enable selection of sows that reach somatic maturity more quickly, give birth to and rear more offspring, and produce more milk [2]. One of the most important factors contributing to favourable production traits in sows is rational feeding during pregnancy and lactation [3, 4]. The quality of the diet of pregnant sows influences programming mechanisms of embryonic and foetal development, the growth, development and health of the mammary gland, and production of colostrum and milk of immunological value, which affects the growth and prenatal development of piglets [5, 6]. One of the most important stages in the pig production cycle is the perinatal period and the piglets’ first week of life. Dietary deficiencies observed in sows at this time adversely affect the offspring, leading to the birth of piglets that are weak and susceptible to disease, increased piglet mortality, and a prolonged fattening period [7, 8]. The capacity of sows to produce a suitable amount of high-quality colostrum and milk, supported by an appropriate diet, not only provides piglets with the nutrients they need, but also stimulates their immunity by supplying antibodies and immunocompetent cells together with the colostrum and milk [9–11]. The immunological quality of the colostrum and milk, which is linked to the diet of sows, is a fundamental factor influencing the health of the offspring [9, 12]. Consumption of low-quality colostrum and milk by piglets reduces or even eliminates lactogenic immunity and increases the frequency of diarrhoea and disturbances of digestion and nutrient absorption associated with colonization of the small intestine by pathogenic bacteria [13, 14]. For many years, these adverse effects were avoided by using antibiotics as feed additives and growth promoters. The ban on the use of antibiotics in pig diets introduced in 2006 has necessitated the development of alternative sow feeding strategies using additives that improve the quality of the colostrum and milk and the growth and health of piglets [15, 16]. Among the feed additives currently used in the diet of pigs, increasing importance is ascribed to probiotics [17, 18].

Literature data indicate that the use of probiotics containing *Enterococcus faecium* in sows increases feed consumption in late gestation and lactation and improves the animals’ body condition [19], counteracting the phenomenon of emaciation in sows during this period. Probiotics containing *Bacillus mesentericus*, *Clostridium butyricum*, and *Enterococcus faecalis* strain, added to feed have also been found to increase sows’ fertility, shorten their oestrus period, and increase piglet birth weight [20]. The use of probiotics *Bacillus subtilis* C-3102 as feed additives in sows during pregnancy and lactation also has a beneficial effect on the composition of the gut microbiota of the piglets [21]. Scharek et al. [22] showed that the use of *Enterococcus faecium* NCIMB 10,415 (SF68) and *Bacillus cereus var*. *Toyoi* bacteria in the diet of sows effectively prevented colonization of the intestines of piglets by *Escherichia coli* strains and led to increased secretion of IgA in the intestines, thereby increasing the defence potential of the mucosa. This reduced the occurrence of diarrhoea, which significantly affects the mortality rate [22, 23].

An important aspect of the effects of probiotics on defence mechanisms in animals is their effect on the quality of sow colostrum and milk [24, 25]. Scharek et al. [22] showed that the use of probiotics as feed additives for sows improved the composition of the colostrum and milk by modifying the composition of the intestinal microbiota and increasing systemic metabolism. However, the most important role of probiotics in terms of animal health and productivity is their capacity to increase the immune potential of the colostrum and milk [26, 27]. Mammary gland secretions physiologically contain numerous antibacterial substances, such as lysozyme and lactoferrin, as well as antibodies, cytokines, growth factors, hormones, and cellular components, which are responsible for immune defence in piglets in the first period of life and stimulate the development of intestinal epithelial cells (IEC), including the gut-associated lymphoid tissue (GALT) system [9, 28–30]. Transfer of specific cellular immunity from the mother to the neonate via the lymphocytes of the colostrum or milk plays an important role in immune system development and in a clinical context [31, 32]. Lymphoid cells ingested with the sow’s milk pass through the intestinal epithelium of piglets and are transported to the mesenteric lymph nodes, where they support the developing immune system by promoting immunoregulation processes [28, 31–33]. About 50% of the cells of sow colostrum and milk are phagocytes (neutrophils, macrophages, and eosinophils), which promote the local immunity of the intestinal mucosa of piglets in the early postnatal period and prevent infections [28].

The health-promoting effects of probiotic preparations depend on the genus, species, and strain of the microorganism used for its production, and their effectiveness can be increased by combining strains acting synergistically [34]. Timmerman et al. [35] showed that probiotics based on numerous strains of the same species as well as preparations composed of strains belonging to different species of microorganisms turn out to be more effective in action because they are active in many places of the intestine in relation to different target cells and show different mechanisms of action. The use of a mixture of *Lactobacillus* spp., *Lactobacillus acidophulus*, *Streptococcus thermophilus*, *Bacillus subtilis*, and *Kluyveromyces fragilis* L-4 uclv strains in pig nutrition reduces the occurrence of gastrointestinal diseases with symptoms of diarrhoea and has a positive effect on the production parameters [36, 37]. Probiotics containing *Lactobacillus* spp., *Bifidobacterium* spp., *Enterococcus* spp., and *Bacillus* spp. reduce the risk of colonization of the intestines with pathogens, and in particular reduce the number of enterotoxigenic *Escherichia coli* and limit the development of *Salmonella* spp in the gut of piglets [36, 38]. Similar effects are exerted by probiotics based on *Lactobacillus sobrius*, *Lactobacillus bulgariccus*, *Saccharomyces thermophilus*, *Saccharomyces boulardii*, and *Pediococcus acidilactici*, which reduce the number of *E. coli* bacteria and regulate the level of IgA in the intestinal mucosa, which affects the local immunity of the intestines and modulates GALT [35, 36, 39]. This group of multi-strain probiotics includes preparations based on EM recommended for use in the diet of pigs, among which EM Bokashi®, product invented by the manufacturer Greenland Technology EM, Janowiec, Poland, is increasingly used. EM Bokashi® contains mixed microorganisms (see Table 2), which are added after the fermentation process of the molasses-based medium, which is an indicator of the high quality of this product and is the basis for its selection for research. Supplementation with EM increases the colonization of the intestinal mucosa by strains of *Lactobacillus* spp. and *Bifidobacterium* spp., at the same time limiting the occurrence of *Salmonella* spp. and *Escherichia coli*, which prevents gastrointestinal diseases with symptoms of diarrhoea in pigs [40]. The use of EM positively influenced the morphological features of the porcine jejunum by increasing the height and width of the intestinal villi, increasing the number of goblet cells and the VH/CD ratio, which is used to assess the degree of digestion of nutrients and the absorption capacity of the small intestine [40, 41]. Moreover, EM Bokashi increased the expression of FABP4, GLUT2, and CLDN1 genes related to gastrointestinal metabolism and raised the level of anti-inflammatory IL10 [40, 42]. This results in an increased absorption of nutrients, weight gain, and, consequently, slaughter efficiency. The action of EM is mainly based on the direct stimulation of GALT cells, which increases the efficiency of immunological processes and leads to a reduction in susceptibility to infection with pathogenic microorganisms, and consequently to an increase in animal health [43]. Our previous research [42] showed that the use of EM as feed additives in the diet of pigs increased the concentrations of certain proinflammatory cytokines, including TNF-α and IL-6, in the colostrum and milk, which increased the protective potential of the colostrum by stimulating cellular immune defence mechanisms. The accompanying increase in the concentrations of IL-4, IL-10, and TGF-β as well as class G and A immunoglobulins in the colostrum and milk of sows receiving EM demonstrates their immunoregulatory effect on Th2 cells, which can lead to an increase in the expression of T regulatory cells and polarization of the immune response from Th1 to Th2.

Thus far, no extensive research has been conducted on the use of EM in sows during pregnancy and lactation and on the effect of EM on the immunological quality of colostrum and milk. The aim of the study was to determine the effect of EM on selected parameters of the specific and nonspecific immune response of sows by assessing the colostrum and milk in terms of the percentage of cells with expression of CD19^+^, CD5^+^CD19^+^, CD21^+^, SWC3a (macrophage/monocyte), and CD11b^+^ molecules on the monocytes and granulocytes as well as the concentrations of lysozyme and acute phase proteins — serum amyloid-A (SAA) and haptoglobin (Hp).

## Materials and Methods

### Experimental Animals and Use of Bokashi® Preparation

All procedures used during the research were approved by the Local Ethics Committee for Animal Testing at the University of Life Sciences in Lublin, Poland (approval no. 55/2013, 15 October 2013).

The study was carried out on a commercial pig farm in Poland. It included 150 sows (Polish Large White × Polish Landrace) at the age of 2–4 years. The sows were kept in individual metal pens with a slatted floor (2.10 m × 0.80 m × 1 m), equipped with feeders and nipple drinkers for water. Two weeks prior to farrowing the sows were placed in a farrowing room, where they were kept in metal pens with a concrete floor covered with litter (2.10 m × 1 m × 1 m). The livestock buildings had automatic climate control and ventilation, with an air flow of 0.567–0.598 m^3^/min, maintaining a maximum temperature of 21.5 °C and relative humidity of 60–65%.

The sows’ diet was based on compound feed produced on-farm — PR-C (complete feed for pregnancy) and PR-L (complete feed for lactation). The composition of the diets is given in Table 1. The animals were fed twice a day and had constant access to water. The size of the daily feed rations, type of feed, and feeding regime were as follows: from insemination to 90 of pregnancy – 3.2 kg/pig/d (PR-C), from days 90 to 107 of pregnancy – 4.0 kg/pig/day (PR-C), from day 108 to farrowing – 3.1 kg/pig/day (PR-L), and from day 2 after farrowing – 2.0 kg/pig/day (PR-L), increased by 0.5 kg/pig/day up to day 7 of lactation. Nutrient content was in accordance with the nutritional recommendations and nutritional values for feed for pigs specified by the NRC [41].

**Table 1:** Composition and nutritional value of sow feed

Ingredient (g)	Pregnant sows (PR-C)	Lactating sows (PR-L)
Barley	36	33
Wheat	29.50	34
Oats	15	10
Triticale	10	-
Soybean meal over 46% HP	3	12
Fermented rapeseed meal	4	4
Fermented soybean meal	-	2
Soybean oil	-	1
Ultramix L.K. Hi Milk 4%	-	4
Ultramix L.P. Hi Breed 2.5%	2.50	-
**Nutritive value**		
Metabolizable energy, MJ/kg	12.60	12.8
Crude protein, g	132	168
Dry matter, g	823	812
Lysine, g	5.73	9.53
Methionine, g	2.17	2.92
Methionine + cystine, g	4.96	5.87
Threonine, g	4.32	6.98
Tryptophan, g	1.48	2.00
Digestible lysine, g	0.472	0.972
Total phosphorus, g	5.08	6.58
Digestible phosphorus, g	2.87	4.31
Calcium,, g	7.02	9.91
Sodium, g	1.75	2.38
Fibre, g	57.8	51.3
Crude fat, g	21.9	30.1
Vitamin A, IU	13,000	12,000
Vitamin D_3_, IU	2000	2000
Vitamin E, mg	117	165

**Fig. 1:**
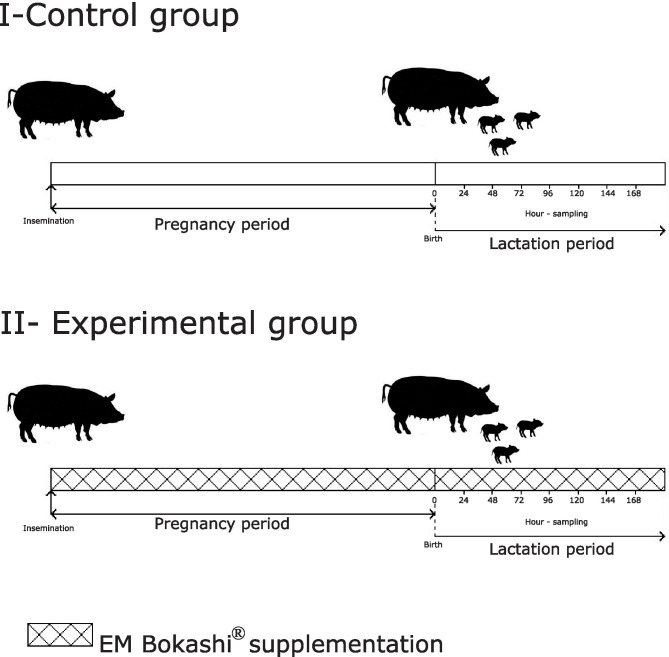
Experience flowchart

Sixty sows (Polish Large White x Polish Landrace) at the age of 2 years, body weight from 160 to 180 kg, and a body condition score (BCS) of 3 according to Charette et al. [44] were randomly selected for the study. The animals were divided into two groups with 30 sows in each, a control group (I) and an experimental group (II). The sows of all groups, during both pregnancy and lactation, were fed according to pig feeding standards specified by the NRC [45] for pregnant and lactating sows on pig farms. The sows in the experimental group (group II), in addition to the basal diet, received a probiotic in the form of the preparation EM Bokashi (batch number 35/27/01/15), developed and manufactured by Greenland Technologia EM (Janowiec, Poland), in the amount of 10 kg/tonne (Fig. 1). The EM Bokashi probiotic used in the experiment contained mixed microorganisms, see Table 2. To avoid loss of viability of the microbial strains in the product, the feed for the control and experimental groups was prepared daily during the entire experiment.

**Table 2:** EM Bokashi composition

	**Microbial composition**	**Strain number**	**Content per gram of product**
1	*Sacharomyces cerevisiae*	Y200007	5 × 10^4^ CFU/g
2	*Lactobacillus casei*	*ATCC 7469*	5 × 10^8^ CFU/g
3	*Lactobacillus plantarum*	*ATCC 8014*	5 × 10^8^ CFU/g
4	*Enterococcus faecalis*	UC-100 (CGMCC No.1.0130)	2.5 × 10^6^ CFU/g
5	*Enterococcus faecium*	NCIMB SF68	5 × 10^9^ CFU/g
6	*Bifidobacterium bifidum*	ATCC 29,521	5 × 10^8^ CFU/g
7	*Bifidobacterium pseudolongum*	ATCC 25,526	5 × 10^8^ CFU/g
8	*Bacillus subtilis*	MA139	4 × 10^11^ CFU/g

Before starting the experimental period, Bokashi preparation was tested in the national reference laboratory of the Department of Hygiene of Animal Feedingstuffs of the National Veterinary Research Institute in Puławy. The tests in the National Reference Laboratory included the assessment of the microbiological purity of the product, i.e. the exclusion of the presence of *Salmonella* spp., and *Escherichia coli*, and determination of the number of lactic acid bacteria, the total number of fungi, and the number of probiotic yeast strains in 1 g of the product (Certificate of analysis—test report no P/15/05555, supplementary materials).

Furthermore, the company manufacturing EM Bokashi preparation evaluated the viability of probiotic bacterial cells and their content per gram of the product and product quality characteristics in their laboratory, thereby guaranteeing that the product used in the experimental group of sows was of the same quality (Quality certificate, supplementary materials). Additionally, the company manufacturing EM Bokashi preparation meets all criteria for food safety and production quality management and has a veterinary number for the product selected for testing α PL 0614002p.

### Collection and Preparation of Colostrum and Milk Samples

Sixty samples of colostrum and milk in a volume of 10 mL were collected in sterile plastic tubes (Medlab Products, Raszyn, Poland). Before sampling, the sows received 1 mL of oxytocin (1 U/mL) intramuscularly. Samples were taken manually from all functional mammary gland packets after disinfection. Colostrum and milk samples were collected from all sows in groups I and II at 0, 24, 48, 72, 96, 120, 144, and 168 h after parturition, beginning with the first piglet born (Fig. 1). The samples were transported to the laboratory at a temperature of + 4–8 °C for no longer than 1 h. Immediately upon delivery to the laboratory, 5 mL of material was designated for cytometric analysis, and the remainder was frozen and stored at −80 °C until further analysis. The samples, in 5 mL volumes, were centrifuged at 1730 × g for 30 min at 4 °C. Then the fat layer and supernatant were separated and removed. The precipitate, containing cells of the mammary gland secretion, was washed twice with 2 mL of RPMI-1640 solution (Biomed, Lublin, Poland) with the addition of 10% foetal bovine serum, antibiotics (penicillin and streptomycin) and an antifungal agent (amphotericin B). After each wash, the samples were centrifuged at 1500 × g for 10 min at 4 °C. After the final centrifugation, the cells were suspended in 2 mL of sterile PBS.

### Determination of Selected Immune Response Parameters

Immune response parameters were determined by flow cytometry. Samples of colostrum and milk were analysed in a flow cytometer (Epics XL, Beckman-Coulter Inc., Brea, California, USA). All determinations were made according to the procedure recommended by the antibody manufacturer.

### Monoclonal and Polyclonal Antibodies

Fluorochrome-labelled monoclonal antibodies for the surface molecules of pig lymphocytes were used for cytometric tests: CD5: FITC (clone 1 H6/8) and CD21: FITC (clone BB6-11C9.6) from Serotec Immunological Excellence (Oxford, England), CD19^+^ (B cells): PE (clone BB6-10A10) from Southern Biotech (Birmingham, Alabama, USA), and SWC3a—Mouse anti Pig Monocyte/Granulocyte:RPE (clone 74–22-15) and Mouse anti Dog CD11b reactivity in pigs (clone CA16.3E10) from Bio-Rad Laboratories, Inc. Life Science Research Group (Hercules, California, United States). The following types of antibodies were used during the negative isotopic control: mouse IgG2a conjugated with FITC, mouse IgG2b conjugated with FITC, and mouse IgM conjugated with PE. Direct cell labelling was used in the study. In each colostrum and milk sample, CD19:PE^+^, CD5:FITC^+^/CD19:PE^+^, CD21:FITC^+^, CD11b:FITC^+^, and Monocyte/Granulocyte (SWC3a):RPE^+^ were determined separately.

### Assessment of the Immunophenotype of Colostrum and Milk Cells of Sows by Flow Cytometry

The immunophenotype of colostrum and milk cells of sows was assessed by the method described by Le Jan [46]. The results were analysed using XL SYSTEM II v.3.0 software and FCS 2.0 format to obtain data in the form of histograms. From 10,000 to 30,000 events were collected in each measurement. Electronic compensation was used to eliminate residual spectral overlaps between individual fluorochromes.

### Acute Phase Protein Analysis in Sow Colostrum and Milk

ELISA kits specific for porcine SAA and Hp (USCN Life Science Inc., Wuhan, China) were used to determine the acute phase protein levels in the sow colostrum and milk, following the manufacturer’s instructions. The absorbance was recorded at 450 nm using an ELISA plate reader (Multiskan RC, Labsystems, Vantaa, Finland). The results (in ng/mL) were multiplied by the appropriate dilution factor and expressed as mg/mL. Each sample was tested in 3 replicates. The results were expressed as mean and standard deviation (± SEM); values of *p* < 0.05 were regarded as significant.

### Determination of the Bacteriolytic Activity of Lysozyme in the Sow Colostrum and Milk

The concentration of lysozyme in the sow colostrum and milk was determined against *Micrococcus luteus* (Serva) by the plate method according to Graham, described by Hankiewicz and Świerczek [47].

### Statistical Analysis

The results were analysed statistically using Statistica 10.0 PL (StatSoft, Krakow, Poland). The analysis included the arithmetic mean and standard deviation (*α* ± SD). Normality was tested with the Shapiro–Wilk test. The significance of differences between means for the control and experimental groups of animals was assessed by the parametric Student *t*-test, and *p* values of less than 0.05 were considered statistically significant (Tables 3 and 4).

**Table 3:** Percentage of subpopulation CD19^+^, CD21^+^, CD5^+^CD19^+^, SWC3a (Monocyte/Granulocyte), CD11b^+^ on granulocyte, and CD11b^+^ on monocyte in the colostrum and milk of sows from groups I and II. Values are expressed as the mean and standard deviation (*α* ± SD)

		**Group I**	**Group II**
**Parameter**	**Hour**	*n* = 30	*n* = 30
**CD19 + (%)**	0	22.05 ± 2.81	43.33 ± 2.41A
	24	29.61 ± 1.62	48.62 ± 3.47A
	48	49.30 ± 2.77A	37.45 ± 2.49
	72	38.40 ± 3.57	34.30 ± 4.41
	96	29.06 ± 3.79	31.58 ± 3.24
	120	15.44 ± 3.18	30.95 ± 3.83A
	144	12.44 ± 3.32	29.23 ± 2.73A
	168	11.16 ± 2.52	28.45 ± 3.31A
**CD21 + (%)**	0	16.89 ± 3.08	33.95 ± 3.68A
	24	25.65 ± 3.76	43.60 ± 3.41A
	48	26.01 ± 1.61	43.89 ± 2.58A
	72	32.57 ± 2.53	41.44 ± 2.86A
	96	26.75 ± 2.03	36.22 ± 3.49A
	120	22.92 ± 1.36	29.92 ± 2.55A
	144	20.49 ± 1.53	31.03 ± 2.39A
	168	20.65 ± 1.61	30.07 ± 4.04A
**CD5 + CD19 + (%)**	0	11.09 ± 2.82	29.39 ± 3.79A
	24	12.78 ± 2.53	31.84 ± 1.86A
	48	23.97 ± 3.76	30.02 ± 1.52A
	72	25.56 ± 2.04	29.33 ± 4.54
	96	24.38 ± 1.53	25.47 ± 4.32
	120	19.89 ± 3.49	24.59 ± 1.18
	144	18.49 ± 4.14	22.50 ± 2.26
	168	18.56 ± 1.53	21.14 ± 2.54
**SWC3a (Monocyte/Graanulocyte) (%)**	0	35.05 ± 4.11	45.46 ± 4.99A
	24	28.01 ± 3.74	53.49 ± 7.11A
	48	25.67 ± 4.25	51.65 ± 7.22A
	72	16.58 ± 2.88	49.04 ± 3.22A
	96	12.34 ± 3.79	47.31 ± 5.26A
	120	9.17 ± 2.54	39.63 ± 4.13A
	144	9.11 ± 2.99	37.54 ± 5.63A
	168	9.25 ± 4.17	35.26 ± 5.12A
**CD11b + on granulocytes (%)**	0	43.32 ± 2.55	49.46 ± 3.91
	24	43.59 ± 4.43	48.40 ± 2.77
	48	39.66 ± 3.81	47.53 ± 2.56A
	72	36.48 ± 4.82	46.35 ± 4.37A
	96	34.08 ± 4.12	42.61 ± 2.81A
	120	33.76 ± 3.67	41.32 ± 4.32A
	144	32.21 ± 2.95	40.42 ± 3.89A
	168	31.34 ± 3.18	39.14 ± 2.64A
**CD11b + on monocytes (%)**	0	42.24 ± 3.88	52.28 ± 3.87A
	24	36.27 ± 5.32	47.72 ± 4.11A
	48	34.62 ± 4.11	42.27 ± 5.03A
	72	33.37 ± 3.87	37.32 ± 5.34
	96	32.35 ± 4.72	36.27 ± 4.85
	120	32.32 ± 4.61	35.07 ± 7.56
	144	30.39 ± 3.73	33.77 ± 3.25
	168	30.28 ± 3.16	32.69 ± 4.22

A indicates a significant increase in the parameter (A *p* < 0.05) between control and experimental group. Values are expressed as the mean and standard deviation (*α* ± SD). I, control group; II, experimental group

**Table 4:** Colostrum and milk concentration of Lysozyme, SAA, and Hp in sows. Values are expressed as the mean and standard deviation (*α* ± SD)

		**Group I**	**Group II**
**Parameter**	**Hour**	*n* = 30	*n* = 30
**Lysozyme (mg/L)**	0	0.12 ± 0.01	0.80 ± 0.01A
	24	0.16 ± 0.02	0.35 ± 0.02A
	48	0.15 ± 0.03	0.35 ± 0.02A
	72	0.27 ± 0.02A	0.15 ± 0.01
	96	0.36 ± 0.02A	0.14 ± 0.02
	120	0.35 ± 0.01A	0.14 ± 0.01
	144	0.40 ± 0.04A	0.14 ± 0.02
	168	0.42 ± 0.01A	0.16 ± 0.03
**SAA (ng/mL)**	0	20.53 ± 1.87	28.32 ± 1.29A
	24	11.69 ± 1.76	40.85 ± 1.81A
	48	12.13 ± 1.69	28.33 ± 1.23A
	72	13.90 ± 0.96	20.87 ± 0.47A
	96	14.53 ± 1.52	19.40 ± 0.73 A
	120	12.35 ± 1.71	18.58 ± 1.09A
	144	10.83 ± 0.78	18.34 ± 0.84A
	168	10.34 ± 1.81	16.50 ± 1.03A
**Hp (ng/mL)**	0	1.46 ± 0.04	1.80 ± 0.03A
	24	0.58 ± 0.04	1.32 ± 0.04A
	48	0.48 ± 0.03	1.26 ± 0.04A
	72	0.38 ± 0.05	1.07 ± 0.03A
	96	0.24 ± 0.01	0.56 ± 0.04A
	120	0.21 ± 0.03	0.50 ± 0.01A
	144	0.20 ± 0.04	0.31 ± 0.02
	168	0.19 ± 0.02	0.26 ± 0.06

A indicates a significant increase in the parameter (A, *p* < 0.05) between control and experimental group. Values are expressed as the mean and standard deviation (α ± SD). I, control group; II, experimental group. Hp haptoglobin, SAA serum amyloid A

## Results

### Evaluation of Flow Cytometry Results

Compared to the control group, the percentages of subpopulation CD19^+^ in colostrum and milk of sows were significantly higher (*p* < 0.05), in group II at 0, 24, 120, 144, and 168 h. However, in the material collected after 48 h, a significant higher percentage (*p* < 0.05) of the CD19^+^ subpopulation was noted in the control group compared to group II.

Compared to the control group, the higher percentages of subpopulation CD21^+^ in colostrum and milk of sows were observed in group II at all sampling hours (0–168).

The significantly higher percentages of the CD5^+^CD19^+^ subpopulation in colostrum and milk of sows compared to the control group were observed in group II at 0, 24, and 48 h.

Compared to the control group, the higher percentages (*p* < 0.05), of subpopulation SWC3a (Monocyte/Granulocyte), in colostrum and milk of sows were observed in group II at all sampling hours (0–168).

The significantly higher percentages (*p* < 0.05), of the CD11b^+^ on granulocytes in colostrum and milk of sows compared to the control group were observed in group II at 48, 72, 96, 120, 144, and 168 g.

The significantly higher percentages (*p* < 0.05), of the CD11b^+^ on monocytes in colostrum and milk of sows compared to the control group were observed in group II only at 0, 24, and 48 h.

### Evaluation of Lysozyme and Acute Phase Proteins in the Colostrum and Milk

A significant higher (*p* < 0.05) concentration of lysozyme in colostrum and milk compared to the control group was observed in group II at 0, 24, and 48 h. However, after 72, 96, 120, 144, and 168 h, the concentration of lysozyme in colostrum and milk was significantly higher (*p* < 0.05), in the control group compared to group II.

Compared to the control group, a significantly higher (*p* < 0.05), concentration of serum amyloid A (SAA) in colostrum and milk was observed in group II at all sampling hours (0–168).

Haptoglobin (Hp) concentration in colostrum and milk of sows in group II was significantly higher compared to the control group at 0, 24, 48, 72, 96, and 120 h.

## Discussion

Processes regulating the immune response of the mammary gland in states of health and disease depend mainly on the population of lymphocytes [48–50]. The dominant population of these cells in colostrum and milk comprises *T* cells, while the percentage of B cells and NK cells is small [51]. Studies by Le Jan [46], Park et al. [52], and Yang et al. [53] show that B cells account for about 20% of the lymphocyte population in colostrum and milk. They are present in mammary gland secretions in the form of virgin cells or memory cells, and co-expression of CD surface molecules on B and T lymphocytes allows them to be transformed into plasma cells producing antibodies. Although B cells are present in colostrum and milk in small numbers, they have high protective potential [54, 55].

There are few studies on the effect of EM-based probiotics administered to females of various species during colostrogenesis on the regulatory and effector functions of B cells in colostrum and milk. Stimulation of the immune system of sows using effective microorganisms has been shown to have a positive effect on lymphocyte selection and maturation in the peripheral lymph nodes and to enrich the colostrum with plasma cells and memory B cells [4]. These observations are confirmed by the present study, in which in the first 2 days postpartum the colostrum and milk of sows receiving Bokashi preparation had a higher percentage of B cells with CD19^+^ expression. The results demonstrate that this probiotic, administered in the form of a feed additive, increased the protective potential of the colostrum and stimulated local humoral mechanisms in the mammary gland, which are responsible for eliminating potential antigens penetrating the organ. Studies in humans showed a higher percentage of CD19^+^ lymphocytes in the colostrum and milk following the use of probiotics as feed additives during colostrogenesis [56, 57]. In contrast, the low percentage of CD19^+^ B cells obtained in our study in the colostrum of sows in the group I, fed a standard diet, is indicative of the low immune potential of the colostrum. The increase in the percentage of B cells shown in the group I between 48 and 72 h postpartum may indicate an infection of the mammary gland appearing at this time, caused by environmental pathogens or those present on the skin of the mammary gland. This seems to be confirmed by results published by Mehrzad et al. [58], who demonstrated activation of B lymphocytes, expressed as an increase in their percentage, following infection of the mammary gland or the progression of subclinical infection into clinical infection. An increase in the percentage of B lymphocytes therefore suggests activation of the humoral response in the initial period of infection of the mammary gland by environmental pathogens. Literature data indicate that this activating effect involving B cells is short-lived and followed by a decrease in the percentage of these cells [4], which was observed in the milk of the control sows from 72 to 168 h postpartum. These dependencies suggest inhibition of the Th2 response, as well as a lack of or inadequate co-stimulation of T and B cells, inhibiting the production of specific antibodies. It should be stressed that the use of Bokashi preparation in pigs as feed additives also stimulates the secretion of IL-4, which activates the humoral immune response [4, 33, 42]. High concentrations of IL-4, IL-10, TGF-β, and class G and A antibodies in the colostrum and milk in conjunction with a high percentage of CD19^+^, CD21^+^, and CD5^+^CD19^+^ cells is indicative of stimulation of B cells and may lead to modification of the immune response towards a Th0/Treg profile, which in turn may contribute to the synthesis of Treg cells maintaining the balance between the Th1 and Th2 responses. These data clearly indicate that the probiotic has an immunoregulatory effect during colostrogenesis in pigs.

Different results were obtained in the present study for the percentage of lymphocytes with expression of the CD21^+^ molecule, which generally appeared on B cells with surface IgD and took part in co-stimulation of lymphocytes taking part in the immune response [59, 60]. Its presence, found exclusively on mature B cells with co-expression of CD19^+^ and CD22^+^, is a condition of a normal humoral response [54]. In the groups I and II, beginning at parturition, a gradual increase in the percentage of CD21^+^ cells was observed in the colostrum. A gradual decrease in the percentage of these cells was noted from 48 h postpartum in the group II and from 72 h in the group I; in the group II, these values were always significantly higher. Given that CD21^+^ is involved in regulating activation and multiplication of B lymphocytes, the increase in the percentage of these cells from 48 and 72 h postpartum is indicative of a reduction in the concentration of specific antibodies in the colostrum and milk. Variation in the expression of the CD21^+^ molecule indicates an immunomodulatory effect of the antigens contained in Bokashi preparation, which act on the entire panel of B lymphocytes and activate them in the mammary gland [58, 59]. The increase in the percentage of CD21^+^ B cells in the group II in combination with the decrease in the percentage of CD19^+^ B cells is indicative of the gradual appearance of memory B cells or is the consequence of activation of Th lymphocytes.

The present study shows that in pigs receiving feed with the addition of Bokashi preparation during pregnancy and lactation, the colostrum, and milk have not only a higher percentage of CD19^+^ and CD21^+^ B cells, but also a high percentage of CD5^+^CD19^+^ lymphocytes. CD5^+^ B cells account for only 5–20% of circulating B cells and are a highly diverse population that can enhance regulation of the immune response and effector cell functions [55, 57]. CD5^+^ B cells therefore promote lymphocyte proliferation and differentiation and the initiation of the Th1 or Th2 immune response. Furthermore, CD5^+^ is an additional signalling molecule able to modify the cellular response to the antigen threshold value; hence, antigen stimulation of the body may induce a multispecific immune response. The additional presence of the CD21^+^ molecule on B cells suggests reactivation of the population of antigen-presenting cells and the secretion of cytokines stimulating B cells to transform into plasma cells and produce antibodies [59–61]. It is worth noting that the percentage of CD5^+^ B cells increased between 24 and 72 h postpartum in the group I, which may be linked to exposure to extrinsic bacterial antigens inducing clinical or subclinical infections of the mammary gland by environmental saprophytic bacteria. Similar observations were made by Appleyard and Wilkie [61] in a study in pigs. In the present study, the percentage of CD5^+^CD19^+^ cells in the colostrum of sows receiving Bokashi preparation was statistically significantly higher between 0 and 48 h postpartum. After this time, it decreased, as in the group I, but remained higher than in the control. The higher percentages of these cells in the group II demonstrate that specific antibodies are produced in pigs and subsequently appear in the mammary gland. The high percentage of these cells noted in the colostrum and milk of sows receiving Bokashi preparation indicates that they may produce Th2 cytokines, mainly IL-10, which acts directly on macrophages and other phagocytes.

The body’s nonspecific immune response, apart from humoral factors, utilizes specialized cells that recognize, ingest, and destroy microbes during phagocytosis [62]. Among cells capable of phagocytosis, the most important are neutrophils, monocytes, and macrophages, which owing to their ability to present antigens to Th lymphocytes and to release cytokines, can additionally take part in the activation and regulation of specific immune response mechanisms [63]. An important role in nonspecific defence is played by the phenomenon of adhesion to endothelial cells, which depends on the expression of an adhesion molecule receptor on the cell surface and is the first stage in the development of inflammation [64]. Increased expression of adhesion molecules, e.g. CD11b/CD18 integrins, may be the effect of stimulation by bacterial lipopolysaccharides and cytokines, such as TNFα [65]. In the present study, samples of colostrum and milk of sows receiving Bokashi preparation had higher SWC3a (monocyte/macrophage) expression than the control samples throughout the experiment. It should be stressed that the high immune potential of colostrum and milk, enhanced by the presence of cells with monocyte/macrophage expression, determines more effective antigen presentation and activation of T and B lymphocytes, which release cytokines and thereby protect piglets against infection in the first few days of life [30]. The present study also showed that CD11b^+^ expression on monocytes and granulocytes was higher in the colostrum and milk of sows receiving Bokashi preparation at all testing times, but statistically significant differences were noted only for monocytes in the first 48 h of the experiment. In both cases, in both the groups I and II, expression of this molecule gradually decreased until 168 h of the experiment, indicating that during this time there was no activation of defence mechanisms involving phagocytes.

One of the important elements of the nonspecific humoral immune response is lysozyme (mucopeptide N-acetylmuramyl hydrolase) [48, 64, 66, 67]. In the present study, the mammary gland secretion of sows receiving Bokashi preparation contained high lysozyme concentrations at the first three testing times, which gradually decreased in subsequent assays. The reverse was noted in the control sows, with a gradual increase in the lysozyme concentration in the colostrum and milk beginning at 72 h of the experiment. These dynamics may be linked to the slow development of inflammatory changes in the mammary gland of sows, which is particularly susceptible to infection with environmental pathogens during lactation. High lysozyme concentrations in the colostrum in the experimental group were correlated with other immune parameters, such as expression of the monocyte/macrophage receptor. High values for both parameters were noted during this period, which may be indicative of lysozyme release from antigen-activated monocytes and macrophages.

In most animal species, including pigs, an important element of assessment of immune system reactivity is the concentration of acute phase proteins [68]. The most important functions of these proteins are restoration of homeostasis in the body through activation of the complement system, a nonspecific reaction associated with opsonization and agglutination, limitation of tissue damage induced by bacteria and lysozyme enzymes released from phagocytic cells, and enhanced chemotactic activity [68, 69]. The present study shows that in terms of diagnostics, it is more efficient to evaluate the concentrations of acute phase proteins in the colostrum and milk of sows than in the serum [4], as it enables detection of conditions such as early inflammation of the mammary gland affecting the health of piglets. The study showed that the Hp concentration in the colostrum and milk of sows from the experimental group throughout the experiment was significantly higher than in the group I and gradually decreased, reaching its lowest value at 168 h postpartum. Similar Hp concentration dynamics were observed in the group I. The results demonstrate that during this period, the mammary gland of sows is well protected against environmental infections, and the Hp concentration noted in the study is the effect of its penetration into the mammary gland from the bloodstream. Contrasting results were found for the concentration of SAA in the colostrum and milk of the sows. In the group II, at 24 h postpartum, the SAA concentration in the colostrum was higher than in the group I, after which it gradually decreased until the end of the experiment. However, throughout the experiment, the concentration of this protein was higher in the group II than in the group I. It is likely that the high concentration of SAA in the colostrum and milk of sows receiving Bokashi preparation is due to stimulation of synthesis of liver proteins by the antigens contained in the probiotic and the accumulation of protein in the liver tissue, from which it passes via the bloodstream to the mammary gland, and SAA takes part only in processes maintaining homeostasis in the body and in mechanisms of innate immunity.

## Conclusions

Supplying piglets with high-quality colostrum and milk in the initial period of life promotes the development of the systemic immune response and the local GALT response, which protect piglets against infection. The study showed that exposure of the pregnant sow to the probiotic microbes contained in EM Bokashi® preparation significantly affects the immunological quality of the colostrum and milk. The numerous microbial strains contained in probiotic formulations activate various cellular processes and act as immunomodulators by increasing macrophage activity, stimulating local antibody synthesis, inducing production of pro- and anti-inflammatory cytokines, and activating NK cells. The use of EM Bokashi® preparation in the diet of pregnant sows caused an increase in the percentage of the subpopulations of B cells with CD19^+^, CD21^+^ and CD5^+^CD19^+^ expression in the colostrum and milk, which demonstrates an increase concentrations of immunocompetent cells and indicates stimulation of humoral immune mechanisms that protect the sow and the piglets against infections by producing activated B cells and synthesizing antibodies. The simultaneous increase in the percentage of cells with SWC3a and CD11^+^ expression, as well as in the concentration of lysozyme and acute phase proteins, indicates intensification of phagocytosis, which protects the body against infection. Further research is necessary for an understanding of the dynamics of the activity of the subpopulations of Th1 and Th2 lymphocytes and regulation of the release of mediators/pro- and anti-inflammatory cytokines within the mammary gland, including the subpopulation of T regulator (Treg) cells, following the use of EM, with regard to increasing the immune potential of the colostrum and milk.

## Data Availability

All data generated or analysed during this study are included in this published article, and are available on request from the corresponding author.
